# Ubiquitous occurrence of a dimethylsulfoniopropionate ABC transporter in abundant marine bacteria

**DOI:** 10.1038/s41396-023-01375-3

**Published:** 2023-01-27

**Authors:** Chun-Yang Li, Michaela A. Mausz, Andrew Murphy, Nan Zhang, Xiu-Lan Chen, Shu-Yan Wang, Chao Gao, María M. Aguilo-Ferretjans, Eleonora Silvano, Ian D. E. A. Lidbury, Hui-Hui Fu, Jonathan D. Todd, Yin Chen, Yu-Zhong Zhang

**Affiliations:** 1 Frontiers Science Center for Deep Ocean Multispheres and Earth System & College of Marine Life Sciences, Ocean University of China, Qingdao, China; 2 State Key Laboratory of Microbial Technology, Marine Biotechnology Research Center, Shandong University, Qingdao, China; 3 Laboratory for Marine Biology and Biotechnology, Pilot National Laboratory for Marine Science and Technology, Qingdao, China; 4 School of Life Sciences, University of Warwick, CV4 7AL Coventry, UK; 5 School of Bioengineering, Qilu University of Technology, Jinan, China; 6 Plants, Photosynthesis and Soil, School of Biosciences, University of Sheffield, Sheffield, S10 2TN UK; 7 School of Biological Sciences, University of East Anglia, NR4 7TJ Norwich, UK

**Keywords:** Biogeochemistry, Marine microbiology

## Abstract

Dimethylsulfoniopropionate (DMSP) is a ubiquitous organosulfur compound in marine environments with important functions in both microorganisms and global biogeochemical carbon and sulfur cycling. The SAR11 clade and marine *Roseobacter* group (MRG) represent two major groups of heterotrophic bacteria in Earth’s surface oceans, which can accumulate DMSP to high millimolar intracellular concentrations. However, few studies have investigated how SAR11 and MRG bacteria import DMSP. Here, through comparative genomics analyses, genetic manipulations, and biochemical analyses, we identified an ABC (ATP-binding cassette)-type DMSP-specific transporter, DmpXWV, in *Ruegeria pomeroyi* DSS-3, a model strain of the MRG. Mutagenesis suggested that DmpXWV is a key transporter responsible for DMSP uptake in strain DSS-3. DmpX, the substrate binding protein of DmpXWV, had high specificity and binding affinity towards DMSP. Furthermore, the DmpX DMSP-binding mechanism was elucidated from structural analysis. DmpX proteins are prevalent in the numerous cosmopolitan marine bacteria outside the SAR11 clade and the MRG, and *dmpX* transcription was consistently high across Earth’s entire global ocean. Therefore, DmpXWV likely enables pelagic marine bacteria to efficiently import DMSP from seawater. This study offers a new understanding of DMSP transport into marine bacteria and provides novel insights into the environmental adaption of marine bacteria.

## Introduction

Approximately eight billion tonnes of the compatible solute dimethylsulfoniopropionate (DMSP) is produced per annum in Earth’s surface waters, by marine algae, bacteria, corals, and some plants [1, 2]. While some DMSP is broken down by the DMSP-producers themselves [3, 4], the vast majority of DMSP is released into marine environments where it is utilized by other heterotrophic microorganisms (mainly marine bacteria) as a carbon, energy, and/or sulfur source [2, 5, 6]. Marine bacteria degrade DMSP to volatile dimethyl sulfide (DMS) and/or methanethiol (MeSH), which play important roles in the global sulfur cycling, with potential consequences for global climate regulation [7, 8].

The SAR11 clade and the marine *Roseobacter* group (MRG) represent two major groups of heterotrophic bacteria in surface ocean waters [9–11] and are active participants in marine carbon, sulfur, and nitrogen cycles [12–14]. Their capacity to metabolize a diverse range of organic matter compounds partially explains their success in different marine habitats [10, 15]. DMSP is ubiquitous in marine environments, with its concentration ranging from nanomolar to several micromolar [2]. Both the SAR11 clade and the MRG are able to utilize DMSP as a nutrient [5, 7], most likely as a source of reduced sulfur, but also of carbon [16]. It has also been reported that intracellular DMSP concentrations can reach 70 mM in the MRG bacterium *Ruegeria pomeroyi* DSS-3 [17], and 180 mM in the SAR11 bacterium *Pelagibacter* HTCC1062 [5], potentially for its antistress properties, as e.g. an osmolyte [2].

DMSP cannot be imported into cells without a specific transporter [18]. To accumulate DMSP to high millimolar intracellular concentrations, efficient transporters are necessary. To date, two transporter families have been reported to import DMSP into cells, the BCCT (betaine, carnitine, and choline transporters) and the ABC (ATP-binding cassette) family [7]. The BCCT transporters are single-subunit transmembrane proteins involved in the uptake of betaine-type organic osmolytes, such as choline and carnitine [19]. DddT, a BCCT transporter whose gene is often found in a cluster with DMSP lyase genes (e.g. *dddD* and *dddX*) in marine DMSP-catabolising bacteria, was reported to import DMSP when heterologously expressed in *Escherichia coli* [20–22]. Other BCCT transporters that imported DMSP and betaine, whose genes were not linked to known DMSP lyase genes, were isolated from the MRG bacteria *Roseovarius nubinhibens* ISM and *Sulfitobacter* sp. EE-36 [21].

ABC transporters, ubiquitous in prokaryotes and eukaryotes, are one of the oldest and largest protein families [23, 24]. In Gram-negative bacteria, they usually consist of three subunits: a periplasmic substrate-binding protein (SBP) responsible for the primary recognition and binding of substrate, a transmembrane domain constituting the translocation pathway, and a cytoplasmic ATP-binding domain to hydrolyse ATP and provide energy for transport. Current knowledge on the ABC transporters which can import DMSP derives mainly from work in *E. coli* and *Bacillus subtilis* [21, 25–27]. For instance, some bacteria, e.g. *Burkholderia ambifaria* AMMD, contain ABC transporter genes in gene clusters with *dddD* and these were also shown to import DMSP in *E. coli* [21]. More recently, physiological experimentation revealed the presence of a high-affinity glycine betaine transporter, likely related to the ABC-type family, capable of importing DMSP in SAR11 bacteria, although the specific identity wasn’t established [28].

All known transporters capable of importing DMSP are multifunctional, i.e., they have promiscuous substrate specificity and are not specific for DMSP [21, 25–28]. Yet, cosmopolitan SAR11 and MRG bacteria, which inhabit oligotrophic regions of the ocean, accumulate high intracellular DMSP concentrations [5, 17]. Thus, we hypothesised that there would be a highly efficient and specific DMSP importer yet to be discovered.

Here, we identified an ABC-type DMSP transporter (DmpXWV) from *R. pomeroyi* DSS-3, the best-characterized model MRG strain which was isolated from coastal seawater [29**–**33], concentrates DMSP to 70 mM and utilizes it as a carbon and sulfur source [7, 31, 34]. DmpX, the SBP of DmpXWV, exhibited a high binding affinity and specificity for DMSP in vitro. Mutagenesis experiments suggested that DmpXWV is a key transporter responsible for DMSP uptake in strain DSS-3. The crystal structure of a DmpX homologue in complex with DMSP was also solved, and the DMSP-binding mechanism of DmpX was predicted. Furthermore, bioinformatic analysis indicated that *dmpX* genes were prevalent in cosmopolitan marine bacteria. This study provides new insights into the environmental adaption of marine bacteria and provides a better understanding of microbial organosulfur cycling.

## Materials and Methods

### Bacterial strains and growth conditions

*Ruegeria pomeroyi* DSS-3 was routinely cultivated in the marine broth medium at 30 °C. The *E. coli* strain BL21(DE3) was grown in the Lysogeny Broth (LB) medium at 37 °C.

### Construction and complementation of Δ*dmpXWV*

To create the Δ*dmpXWV* knockout mutant regions of homology at the 5′ and 3′ ends of *dmpXWV* were amplified, alongside the gentamicin cassette from the p34s-Gm plasmid. Amplified DNA fragments were assembled into linearised pk18mob*sacB* using the HiFi assembly kit (New England Biolabs) according to the manufacturer’s instructions. To construct the complementation plasmid, *dmpXWV* was cloned into linearised pBBR1McS-km using the HiFi assembly kit. Plasmids were electroporated into competent *Escherichia coli* S17.1 λpir and conjugated into *R. pomeroyi* DSS-3. The knockout mutant was selected onto minimal media plates containing gentamicin (10 µg/ml), with resultant colonies screened for kanamycin resistance. Kanamycin-sensitive clones were PCR screened and products sequenced to confirm a double crossover. The complemented mutant strain was selected on MB plates containing gentamicin (10 µg/ml) and kanamycin (80 µg/ml), and colonies confirmed by PCR screening and sequencing.

### Gene cloning, point mutation and protein expression and purification

The genes encoding SBPs from *R. pomeroyi* DSS-3 or *R. nubinhibens* ISM were amplified from the genome of *R. pomeroyi* DSS-3 or *R. nubinhibens* ISM by PCR using *FastPfu* DNA polymerase (TransGen Biotech, China). The *dmpX* homologue from *P*. sp. strain HTCC7211 (locus tag HTCC7211_00013840) was synthesized by the Beijing Genomics Institute (China). The related genes were then cloned into the *Nde*I/*Xho*I restriction sites of the pET-22b vector (Novagen, Germany) with a C-terminal His tag. Point mutations in DmpX were introduced using the PCR-based method and verified by DNA sequencing. The SBPs and corresponding mutant forms were produced in *E. coli* BL21 (DE3). The cells were cultured in the LB medium with 0.1 mg/ml ampicillin at 37 °C to an OD_600_ of 0.8–1.0 and then induced at 18 °C for 16 h with 0.5 mM isopropyl-β-D-thiogalactopyranoside (IPTG). After induction, cells were collected by centrifugation, resuspended in the lysis buffer (50 mM Tris-HCl, 100 mM NaCl, 0.5% glycerol, pH 8.0), and lysed by a pressure crusher. The proteins were first purified by affinity chromatography on a Ni^2+^-NTA column (GE Healthcare, US), and then fractionated by gel filtration on a Superdex G75 column (GE healthcare, US).

### Isothermal titration calorimetry measurements

Isothermal titration calorimetry (ITC) measurements were performed at 25 °C using a MicroCal iTC200 system (GE Healthcare, US) or MicroCal PEAQ-ITC system (Malvern Panalytical, UK). The sample cell was loaded with 280 μl of protein sample (30–100 μM), and the reference cell contained distilled water. The syringe was filled with 40 μl of substrates (100 μM–1 mM). The proteins and substrates were kept in the same buffer containing 10 mM Tris-HCl (pH 8.0) and 100 mM NaCl.

### Crystallization and data collection

The purified *Rn*DmpX protein was concentrated to 7 mg/ml in 10 mM Tris-HCl (pH 8.0) and 100 mM NaCl. To obtain crystals of the *Rn*DmpX/DMSP complex, *Rn*DmpX protein was mixed with DMSP in a 1:5 molar ratio. Initial crystallization trials for the *Rn*DmpX/DMSP complex were performed at 20 °C using the sitting-drop vapor diffusion method. Diffraction quality crystals of the *Rn*DmpX/DMSP complex were obtained in hanging drops containing 0.2 M magnesium chloride, 0.1 M Bis-Tris (pH 5.5), and 25% (wt/vol) polyethylene glycol 3350 at 20 °C after 2-weeks incubation. Crystals of the *Rn*DmpX/DMSP complex soaked with 5-amino-2,4,6-triiodoisophthalic acid (I3C) were obtained by soaking the *Rn*DmpX/DMSP complex with I3C compound according to the protocol of the I3C Phasing Kit (Hampton Research, US). Diffraction quality crystals of the *Rn*DmpX/DMSP complex were obtained in hanging drops containing 0.2 M calcium chloride, 0.1 M Hepes (pH 7.0), and 20% (wt/vol) polyethylene glycol 6000 at 20 °C after 2-weeks incubation. X-ray diffraction data were collected on the BL19U1 and BL17U1 beamlines at the Shanghai Synchrotron Radiation Facility. The initial diffraction data sets were processed with the HKL3000 program [35].

### Structure determination and refinement

The crystals of the *Rn*DmpX/DMSP complex and the *Rn*DmpX/DMSP complex soaked with I3C belong to the *P*4_1_ space group. The structure of the *Rn*DmpX/DMSP complex soaked with I3C was determined by single-wavelength anomalous dispersion phasing. The crystal structure of the *Rn*DmpX/DMSP complex was determined by molecular replacement using the CCP4 program Phaser [36] with the structure of the *Rn*DmpX/DMSP complex soaked with I3C as the search model. The refinements of these structures were performed using Coot [37] and *Phenix* [38]. All structure figures were processed using the program PyMOL (http://www.pymol.org/).

### Circular dichroism (CD) spectroscopy

CD spectra for WT *Rn*DmpX and its mutants were carried out in a 0.1 cm-path length cell on a JASCO J-810 Spectrometer (Japan). All proteins were adjusted to a final concentration of 30 μM in 10 mM Tris-HCl (pH 8.0) and 100 mM NaCl. Spectra were recorded from 250 to 200 nm at a scan speed of 200 nm/min.

### ^14^C-DMSP synthesis and determination of radioactive and molar concentration

^14^C-DMSP ([1-^14^C]DMSP) was synthesised from ^14^C-acrylic acid and dimethylsulfide as described earlier [20] and diluted at a 1:10 (vol/vol) ratio with ^12^C-DMSP. Triplicates of 1 µl ^14^C:^12^C-DMSP in 3 ml scintillation fluid (EcoLume Liquid Scintillation Cocktail, MP Biomedical, US) were measured on a liquid scintillation analyzer (Tri-Carb 2800TR, Perkin Elmer, UK) after overnight equilibration. The radioactive concentration of the ^14^C-DMSP stock thus was determined to be 0.448 kBq µl^-1^.

To confirm the identity and concentration of ^14^C:^12^C-DMSP, we used a published LC-MS approach for nitrogen-containing osmolytes quantification [39] due to the structural similarity of the nitrogen-osmolyte glycine betaine and the sulfur-containing DMSP. The radiolabelled DMSP-stock was diluted 10^-6^ times in methanol:chloroform: water (12:5:1, vol/vol) and 200 nM d_11_-glycine betaine (Cambridge Isotope Laboratories Inc., US) added as internal standard (ISTD) to analytical triplicates. Sample separation was performed using a Discovery HS F5 column and HS F5 Supelguard column (both Supelco, US) on a Dionex 3400RS HPLC coupled to an amaZon SL ion trap mass spectrometer (Bruker, UK) for mass detection via Electrospray ionisation in positive ion mode. In parallel, triplicate standards covering a range of 0.005-2 µM DMSP (dimethylpropiothetin hydrochloride, Supelco) were prepared in methanol:chloroform: water (12:5:1, vol/vol), spiked with 200 nM d_11_-glycine betaine ISTD and measured by HPLC-MS to obtain a calibration curve [40]. ^12^C-DMSP was detected at *m/z* 135 and the ISTD at *m/z* 129 using Bruker QuantAnalysis software to determine the ratio of the peak area for ^12^C-DMSP to the ISTD in each sample. The molar concentration of ^12^C-DMSP in the ^14^C:^12^C-DMSP stock was found to be 328.8 ± 15.2 mM. Since ^14^C could not be detected, the ratio of ^14^C:^12^C might have been lower than the assumed 1:10 (vol/vol) ratio.

### Uptake of ^14^C-labeled DMSP

A single colony of *R. pomeroyi* DSS-3 WT, Δ*dmpXWV* mutant, or complemented Δ*dmpXWV* mutant was inoculated into 5 ml of marine broth medium (MB) and grown overnight at 30 °C under constant shaking (170 rpm). 1 ml preculture was inoculated into 50 ml MB and grown at 30 °C with shaking (130 rpm) until an OD_540_ of ~0.5-0.7 was reached, then used for DMSP uptake assays.

Due to a relatively low radiochemical and mid-millimolar range molar concentration of the ^14^C:^12^C-DMSP stock, we could not conduct uptake kinetics experiments. Instead, we used three DMSP concentrations (50, 150, and 300 µM) and added them to 3 ml of cultures in biological triplicates. Cells were incubated at 30 °C with shaking (130 rpm) for 5 min, filtered onto 0.2 µm pore size Supor filters (∅ 25 mm, Pall Corporation, US), and washed 3x with 1 ml MB medium. Wet filters were transferred to 6 ml scintillation vials and 3 ml EcoLume Liquid Scintillation Cocktail added. After equilibration overnight, samples were measured on a Tri-Carb 2800TR liquid scintillation analyzer. Cells fixed in 2% (vol/vol) freshly prepared paraformaldehyde solution for >15 min at 4 °C in the dark prior to radioisotope addition were used for background correction. Uptake rates were normalized by cell number. A Shapiro-Wilk test was used to test for normal distribution of uptake rates and a two-way ANOVA with Tukey’s multiple comparison test to analyse whether uptake rates of the wild-type and mutant cultures at the three different DMSP concentrations were significantly different.

### Flow cytometry

Bacterial cell numbers used for normalization of uptake rates were determined by flow cytometry [41]. 1 ml culture was fixed with a final concentration of 0.5% (vol/vol) glutaraldehyde (Electron microscopy grade, BDH, AE) for 30 min at 4 °C, snap frozen in liquid nitrogen, and stored at –80 °C. Samples were thawed at 37 °C for 5 min, stained with SYBR gold (Invitrogen, US) (final concentration 10^-4^ of commercial stock) for 10 min at 60 °C in the dark. 75 to 200-fold sample dilutions in sterile TE buffer (10 mM Tris-HCl, 1 mM EDTA, pH 8.0) were counted in analytical triplicates on a CytoFLEX flow cytometer (Beckman Coulter, UK) at a flow rate of 30 µl min^-1^ for 60 sec. The instrument was equipped with a 50 mW 488 nm solid-state diode laser with standard filters and the discriminator set to 525 nm (green fluorescence) (Fig. S1).

### Bioinformatic analysis

**Fig. 1:**
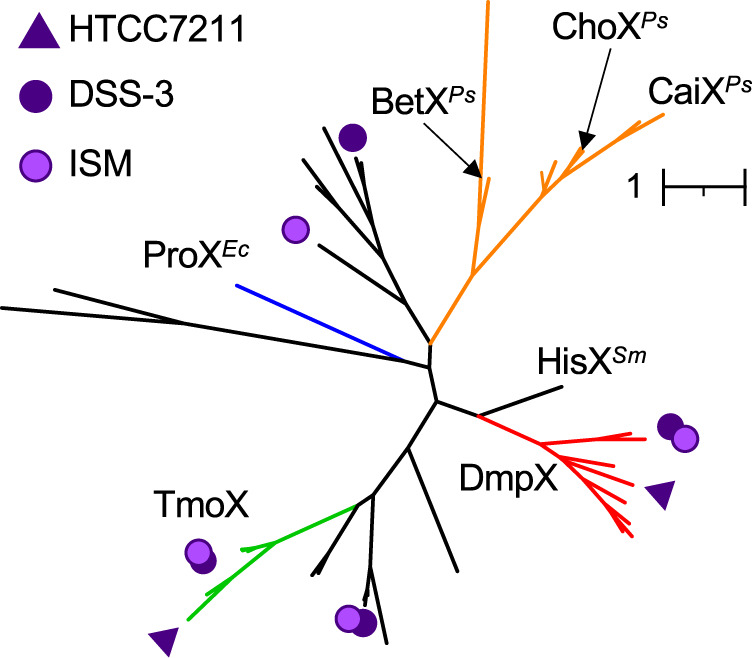
Phylogeny of all identified SBPs containing the pfam04069 domain in selected marine bacteria related to the MRG (strains *Ruegeria pomeroyi* DSS-3; *Roseovarius nubinhibens* ISM) and the SAR11 clade (*Pelagibacter* sp. HTCC7211), as well as selected bacteria including *Escherichia coli* (Ec), *Pseudomonas syringae* (Ps), *Sinorhizobium meliloti* (Sm) that possess experimentally characterised SBPs with affinity for closely related DMSP analogue. These include TMAO-TmoX, choline-ChoX, carnitine-CaiX, proline betaine-ProX, glycine betaine-BetX, and histidine-HisX.

All phylogenetic reconstructions were performed using IQTREE and tress were edited to determine phylogenetic relationships between DmpX and other SBPs encoding the pfam04069 (OpuAC) domain, we used the IMG/JGI database and online bioinformatics server (https://img.jgi.doe.gov). Strains (*n* = 8) previously shown to possess functionally characterised representatives of this SBP family were searched for any ORFs containing the pfam04069 domain (Function search, ‘filter’ = pfam[list]), see Table S1 for strains and genes used for Fig. 1. Next, genomes from bacterial isolates, MAGs and SAGs (n = 8730) related to phylogenetically distinct marine bacteria (cyanobacteria and heterotrophs) and archaea, deposited in IMG/JGI (as of 29^th^ November 2022), were scrutinised for the presence of any ORFs encoding the pfam04069 (OpuAC) domain (Function search, ‘filter’ = pfam[list]). 8277 ORFs from ~3400 genomes were identified and downloaded. Using a local version of blast+(v2.10.0), these ORFs were screened by BLASTP (stringency e^-50^; minimum similarity 30%) using strain DSS-3 as the query sequence, returning 1427 ORFs (Table S2). A phylogenetic tree was reconstructed, using TmoX as the outgroup. Any sequences clustering outside the DmpX major clade were removed from the data. Using HMMER v3.3 (hmmbuild), we generated a manually curated profile hidden Markov model (pHMM) using sequences falling within the DmpX clade and possessing all key residues. Based on our previous research [13, 42], we also generated a pHMM for TmoX, a highly synthesised SBP in the ocean [13].

The *Tara* Oceans metagenomes (OM_RGC_v2_metaG) and metatranscriptomes (OM_RGC_v2_metaT) were searched using these pHMMs according to a published method [43]. Briefly, the Ocean Gene Atlas web interface [44] was used to extract sequences and abundances, using a stringency of e^–70^. Sequence abundances were normalized using the median abundance of 10 single-copy marker genes [45], each with a stringency of e^–40^, in both metagenomes and metatranscriptomes. Normalized abundances per site were summed by phylogeny at the level of Order, if available, and by the lowest available taxonomic rank above that if not available.

## Results

### Identification of a potential DMSP specific ABC transporter

*R. pomeroyi* DSS-3 was used as the model to identify the presence of ABC transporters involved in DMSP uptake since this bacterium is well known to grow on DMSP and it accumulates DMSP at 70 mM intracellular levels [17, 31]. We first screened the *R. pomeroyi* DSS-3 genome for genes encoding SBPs containing a pfam04069 domain. This substrate binding domain is found in a number of characterized SBPs that are able to transport DMSP and other related molecules, such as glycine betaine, choline, carnitine and trimethylamine *N*-oxide (TMAO) [13, 46]. This analysis identified four open reading frames (SPO1131, SPO1548, SPO2441 and SPOA0231) encoding SBPs (Fig. 1), including the previously characterized TMAO-specific SBP, TmoX (encoded by SPO1548) that does not show any binding affinity to DMSP [13, 42], and the SPOA0231 that had ~70 % amino acid identity to a gene product from *Rhodobacteriales* bacterium KLH11, whose gene is in a gene cluster with *dddD* [21].

**Fig. 2:**
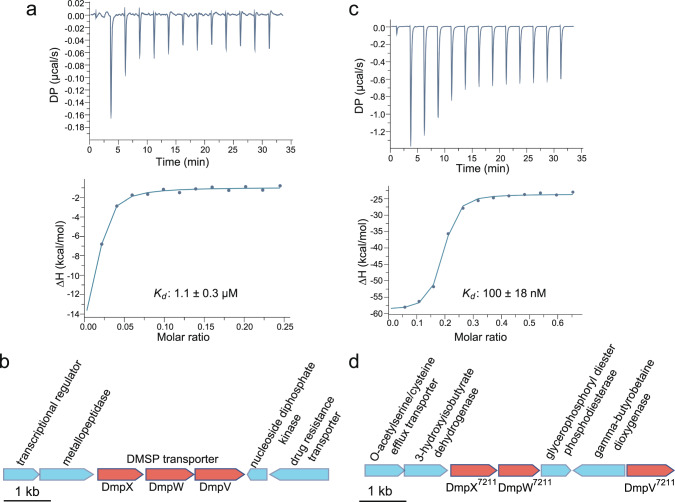
Characterization of the DmpX protein and the genomic position of *dmpXWV*. **a** ITC data for titrations of DMSP into recombinant DmpX from *R. pomeroyi* DSS-3. **b** The DMSP transporter gene cluster in the genome of *R. pomeroyi* DSS-3. **c** ITC data for titrations of DMSP into recombinant DmpX from *Pelagibacter* sp. HTCC7211. **d** The DMSP transporter gene cluster in the genome of *Pelagibacter* sp. HTCC7211.

To verify whether the other three ORFs encoding SBPs (i.e. SPO1131, SPO2441 and SPOA0231) could bind DMSP, we overexpressed them in *E. coli*, purified the recombinant proteins, and measured their affinities for DMSP using isothermal titration calorimetry (ITC). The recombinant SBP, encoded by SPO2441, possessed a high binding affinity for DMSP, with a *K*_*d*_ (dissociation constant) of 1.1 μM (Fig. 2a), while the other two recombinant SBPs (encoded by SPO1131 and SPOA0231) did not. Hereafter we designate the SBP encoded by SPO2441 as DmpX, with Dmp standing for Dimethylsulfoniopropionate. To analyse the substrate specificity of DmpX, we tested the affinities of this SBP for DMSP analogues, including TMAO, betaine, choline, and carnitine using ITC. Compared to DMSP, DmpX presented little binding affinity for TMAO, betaine, choline, and carnitine (Table S3), suggesting that DmpX is highly specific for DMSP.

In the *R. pomeroyi* DSS-3 genome*, dmpX* is located in a gene cluster next to ORFs encoding a transmembrane subunit (SPO2442) and an ATP-binding subunit (SPO2443) of the ABC transporter (Fig. 2b). Usually, the SBP confers the substrate specificity of the ABC transporter [13, 46], which suggests that SPO2441-2443 encode a transporter specific for DMSP. Hereafter we designate SPO2441-2443 as genes encoding DmpX, DmpW, and DmpV, respectively. It is noteworthy that a DmpX-like protein is coded for in the genome of SAR11 strain *Pelagibacter* sp. HTCC7211 (WP_034399263.1), which shared ~35% amino acid identity with *R. pomeroyi* DSS-3 DmpX. To determine whether this SAR11 SBP (hereafter designated DmpX^7211^) is also capable of binding to DMSP, we chemically synthesized the gene, overproduced the recombinant SBP in *E. coli*, and purified it prior to ITC titration. DmpX^7211^ also had a high binding affinity for DMSP, with a *K*_*d*_ of 100 nM (Fig. 2c, d). Together, our data suggest that these model MRG and SAR11 clade bacteria encode a high-affinity ABC transporter for DMSP uptake.

### The importance of *R. pomeroyi* DSS-3 DmpXWV in the import of DMSP

**Fig. 3:**
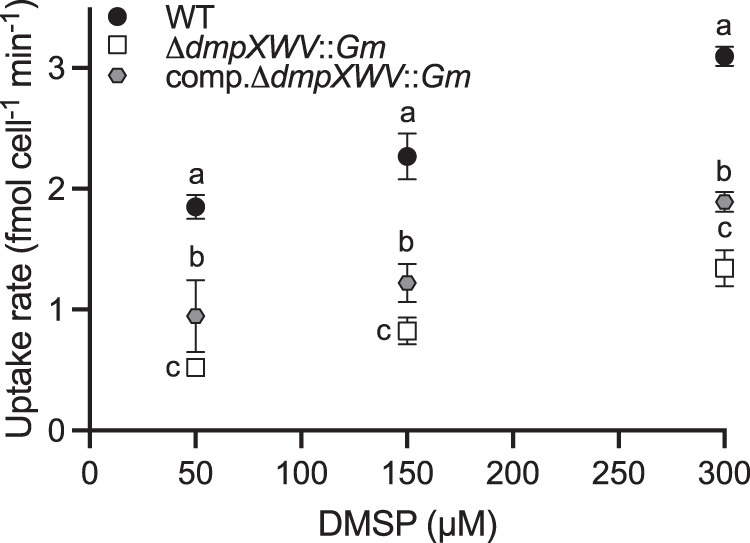
DmpXWV is important for the uptake of DMSP in *R. pomeroyi* DSS-3. ^14^C-DMSP uptake in the WT, Δ*dmpXWV*::*Gm* mutant, and complemented mutant (comp. Δ*dmpXWV*::*Gm*) at three concentrations by spiking DMSP in the marine broth medium. The error bar represents the standard deviation of triplicate experiments. Small letters indicate significant differences (*p* < 0.05) between wild-type, mutant and complemented mutant cultures at different DMSP concentrations based on a two-way ANOVA with Tukey’s multiple comparison test.

To verify the function of *dmpXWV* in vivo, a Δ*dmpXWV*::*Gm* mutant was constructed, which disrupted the genes encoding the entire *dmpXWV* gene cluster. To uncover the role of *dmpX* in DMSP transport, we performed a ^14^C-radioisotope uptake experiment using ^14^C-DMSP and compared ^14^C- uptake at three concentrations (50, 150, and 300 μM). Uptake of ^14^C-DMSP in the mutant Δ*dmpXWV*::*Gm* significantly decreased in comparison to the wild-type (WT) (*p* < 0.001) with the strongest reduction at 50 μM (~5 times) and the lowest decrease at 300 μM (~3 times) (Fig. 3, Table S4). Complementation with the *R. pomeroyi dmpXWV* gene did partly restore ^14^C-DMSP import (Fig. 3). These uptake patterns support that *dmpXWV* is a DMSP transporter although it is clear that other DMSP transporters also exist in this bacterium.

### The crystal structure and DMSP-binding mechanism of DmpX

**Fig. 4:**
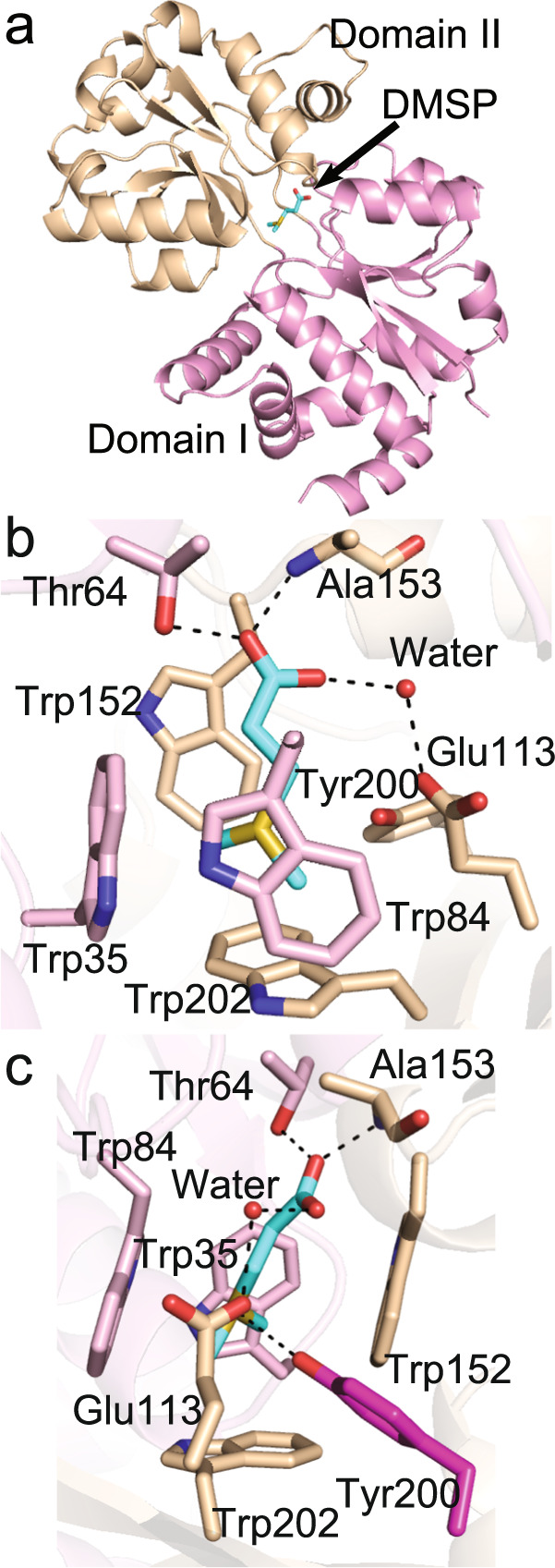
Structural analysis of the *Rn*DmpX/DMSP complex. **a** Overall structure of the *Rn*DmpX/DMSP complex. Domain I is coloured in pink, and domain II in wheat. The location of the DMSP molecule is indicated by a black arrow. **b** Residues composing the DMSP-binding pocket. The oxygen, nitrogen and sulfur atoms in the structure are coloured in red, blue and yellow, respectively. The carbon atoms of residues from domain I are shown in pink, and from domain II in wheat. The carbon atoms of the DMSP molecule are shown in cyan. **c** Structural analysis of the function of residue Tyr200 (carbon atoms coloured in purple) in binding DMSP.

To study the DMSP-binding mechanism of DmpX, we attempted to solve the crystal structure of DmpX in the presence and absence of DMSP. While DmpX proteins of *R. pomeroyi* DSS-3 and *Pelagibacter* sp. HTCC7211 did not yield crystals, a DmpX homologue from MRG strain *Roseovarius nubinhibens* ISM, *Rn*DmpX, sharing 58% amino acid identity with *R. pomeroyi* DSS-3 DmpX, yielded good quality crystals when co-crystallization with DMSP was conducted. The recombinant *Rn*DmpX also presented a high binding affinity for DMSP, with a *K*_*d*_ of 474 nM (Fig. S2), and the crystal structure of *Rn*DmpX in complex with DMSP was solved (Table S5). *Rn*DmpX comprises two domains connected by two hinge regions (Val106-Gly111 and Arg244-Val247), with a DMSP molecule bound between the two domains (Fig. 4a).

The binding pocket of *Rn*DmpX is composed of Trp35, Thr64 and Trp84 from domain I, and Glu113, Trp152, Ala153, Tyr200 and Trp202 from domain II (Fig. 4b). Residues Thr64 and Ala153 participate in binding DMSP through hydrogen bonds with the carboxyl group of DMSP (Fig. 4b). Glu113 also participates in binding DMSP through a water-mediated hydrogen bond with DMSP (Fig. 4b). The side chains of the four tryptophan residues form a hydrophobic cage to accommodate the tertiary sulfonium group of DMSP (Fig. 4b). In particular, we found that Tyr200 could form a hydrogen bond with the positive-charged sulfur of DMSP (Fig. 4c). The DMSP binding mode of *Rn*DmpX shares similarities with those reported in DMSP lyases [47, 48]. However, to the best of our knowledge, the hydrogen bond interaction between residues and the sulfur atom of DMSP has not been reported before. When Tyr200 was mutated to Ala or Phe, the binding affinities of *Rn*DmpX for DMSP were abolished (Fig. S3), suggesting that this interaction plays an important role in DMSP binding. Circular-dichroism (CD) spectroscopy analysis showed that the secondary structures of the mutants exhibit little deviation from that of WT *Rn*DmpX (Fig. S4), indicating that binding affinity loss was caused by amino acid replacement rather than by structural change. Residues involved in binding DMSP are highly conserved in MRG and SAR11 clade (Fig. S5), suggesting that the binding mechanism of DmpX in these marine bacteria may be similar to that in *R. nubinhibens* ISM.

### Distribution of DmpXWV in marine bacteria through comparative genomics and metagenomics/metatranscriptomics

**Fig. 5:**
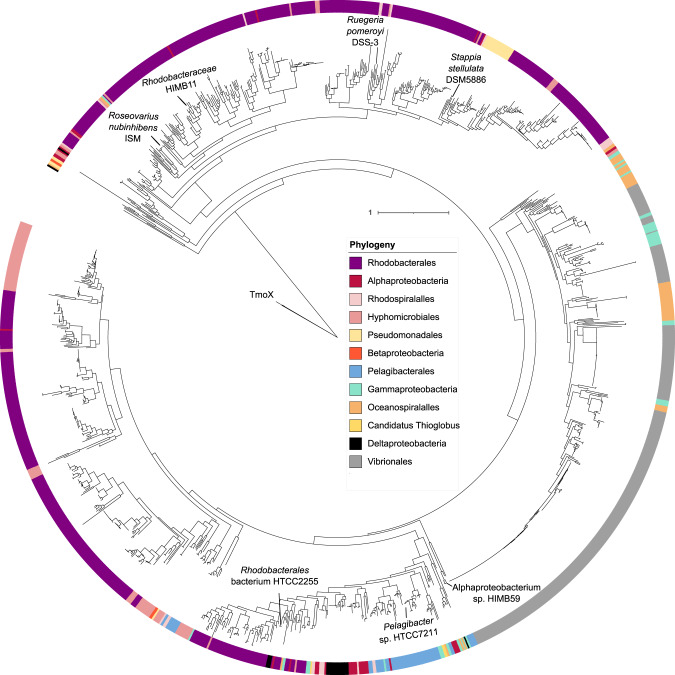
Environmental distribution and expression of *dmpX* in the global ocean. Phylogenetic reconstruction of DmpX based on sequences retrieved from genomes of bacterial isolates, MAGs and SAGs. Blue and Red circles represent *Roseobacter* and SAR11 DmpX homologues, respectively, that were experimentally validated in this study or those shown in figure S5.

To determine how widespread and potentially important *dmpXWV* is in marine bacteria, we scrutinised the genomes of both cultivated isolates, metagenome-assembled genomes (MAGs) and single-amplified genomes (SAGs) associated with the marine environment. Using the IMG/JGI database, genomes were scrutinised for the presence of *dmpX* and a phylogenetic tree of the gene products was reconstructed using TmoX, the SBP for TMAO, as the outgroup [13]. We found an unexpected diversity and occurrence of DmpX, revealing vast expansion outside the MRG and SAR11 clade (Fig. 5). This included occurrence in other abundant *Alphaproteobacteria* (SAR116, unclassified), *Gammaproteobacteria* (*Candidatus* Thioglobus, *Oceanospirillales*), and *Deltaproteobacteria* (SAR324). As seen with other abundant SBPs identified in the ocean, such as TmoX [13] and AepX [43], two distinct polyphyletic clades were apparent, which is predominantly partitioned by lifestyle (free-living versus particle-associated). *Rn*DmpX and those from clade I (including DmpX^7211^) and clade II (including DSS-3) all shared conserved key residues, suggesting a common mechanism was evident across the diversity of DmpX.

**Fig. 6:**
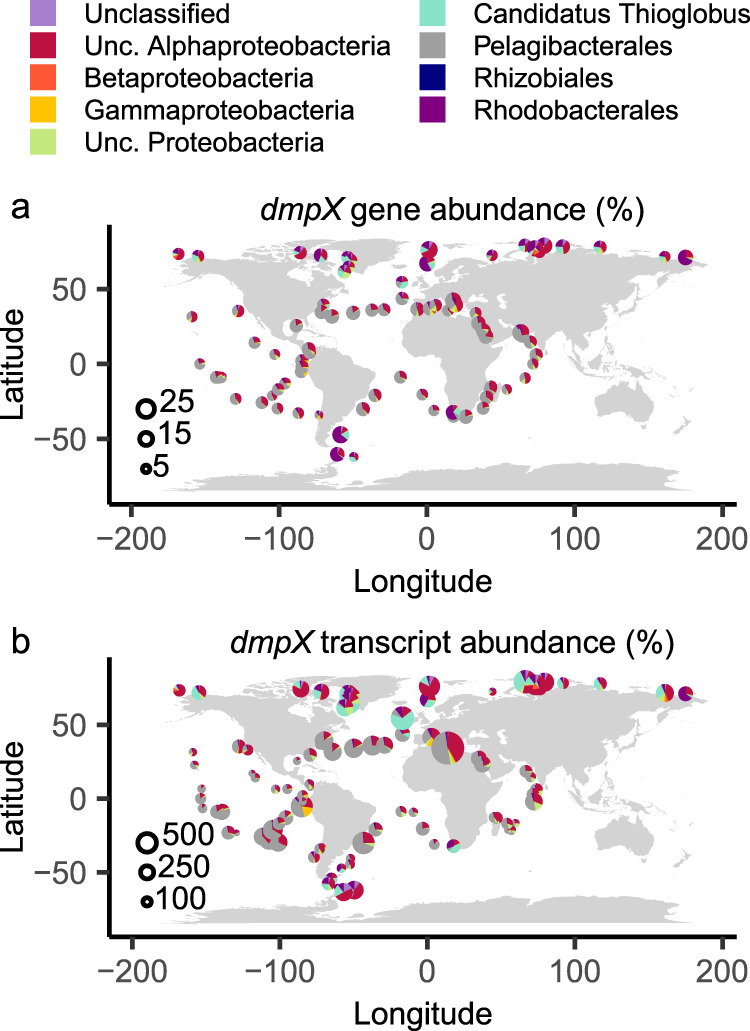
The distribution, diversity, and expression of *dmpX* across the global ocean. *dmpX* gene (**a**) and transcript (**b**) abundance by taxonomic assignment in marine surface waters. Normalized gene and transcript abundance in the *Tara* Ocean dataset (surface water sites only). Circle radius represents normalized abundance, calculated as a percentage of the median gene abundance or transcript abundance of 10 single-copy marker genes. Relative abundance of different taxa at each site is shown as pie charts, summing taxonomy at the level of Order, or the lowest taxonomic rank above Order. In the figure legend, unclassified is abbreviated to Unc., for clarity of presentation.

Based on this unexpected diversity, we investigated the abundance and phylogeny of *dmpX* in the global ocean metagenomes and metatranscriptomes. We searched the *Tara* Oceans metagenomic and metatranscriptomics datasets for homologues, using manually curated profile hidden Markov models (pHMMs). Homologous sequences retrieved from our searches were manually examined to ensure essential residues were conserved, and to determine whether DmpX and closely related SBPs i.e. TmoX, could be easily distinguished (Fig. 5). Normalized read abundances were combined with phylogenetic assignments to determine the prevalence of *dmpX* at surface water sampling sites (Fig. 6). The majority of marine *dmpX* genes and transcripts were closely related to *Pelagibacterales*, with unclassified *Alphaproteobacteria* being the next most abundant group. However, sequences belonging to *Rhodobacterales* as well as the gammaproteobacterium *Candidatus* Thioglobus were abundant at sampling sites in the polar oceans (Fig. 6). In certain oceanic regions the relative abundance of *dmpX* transcripts was significantly higher (*dmpX* copies were up to 700% of median single-copy core gene transcript levels) than its corresponding gene abundance (up to 25% of cells contained *dmpX*), suggesting either DMSP catabolism is disproportionately higher in certain regions, or there are other as yet unidentified transporters active.

## Discussion

The microbial use of DMSP as osmolyte or nutrient via DMSP catabolism, are globally important biogeochemical processes [7]. Critically, these processes require the import of DMSP into cells in the heterotrophic and/or phototrophic organisms that do not make DMSP themselves [2, 49]. The MRG and SAR11 clade, two dominant groups in marine surface waters, are key DMSP catabolic bacteria, and thus important drivers of marine carbon and sulfur cycling [5, 7, 17]. However, the importance of these two groups using DMSP as a carbon source was recently questioned [16]. Here, bioinformatics was used to identify ABC transporters with the potential to import DMSP from the genome of the model MRG bacterium *R. pomeroyi* DSS-3. Of four candidate transporters only one, termed DmpXWV, had high binding affinity and specificity for DMSP and this played a key role in DMSP import in *R. pomeroyi* DSS-3 – with a *dmpXWV*^-^ mutant significantly losing DMSP import activity compared to wild type strains (Fig. 3). Other potential transporters in *R. pomeroyi* DSS-3, e.g. a DddT-like protein (SPO3186), bioinformatically predicted are likely responsible for the low level DMSP import seen in the *dmpXWV*^-^ mutant (Fig. 3).

All the previously reported transporters capable of importing DMSP are likely multifunctional [21, 25–28]. In addition to DMSP, they also imported varied quaternary ammonium compounds (QACs), such as betaine, carnitine, and choline [21, 25–28]. This may be advantageous allowing them to multitask and import varied osmolytes/nutrients when they become available in oligotrophic and or changing settings. However, given that DMSP is ubiquitous in marine environments where sulfur is abundant whilst nitrogen is generally more scarce [28], one would predict the capacity to import DMSP is likely very important for marine bacteria and, thus, such organisms would require a specific DMSP importer. Indeed, biochemical data here suggests that DmpX is one such highly specific DMSP importer found in diverse marine bacteria. Note, we cannot exclude that DmpXWV does not import other compounds structurally related to DMSP into cells in vivo and growth experiments of the *dmpXWV* deletion mutant on a wider range of DMSP analogues would help to provide further insights. However, our biophysical data presented here demonstrates that the DmpX homologues tested here cannot bind other substrates at environmentally relevant concentrations.

Within many bacteria that cleave DMSP and grow on its catabolites as a source of carbon, their DMSP lyase genes *dddD* or *dddX* are often linked with a transporter gene, either *dddT* or an ABC transporter gene cluster [16, 21, 22]. However, most other DMSP lyase and catabolic genes involved in the demethylation pathway genes are not commonly located in a gene cluster with potential DMSP transporters. The genes encoding DmpXWV identified here are also not colocalised with other known DMSP catabolic genes (Fig. 2b). Bioinformatic analysis revealed *dmpXWV* was prevalent in abundant marine bacteria, including the MRG and the SAR11 clade but also found in cosmopolitan *Gamma-* and *Deltaproteobacteria*. Furthermore, high transcript levels of *dmpX* were detected across the global ocean, with certain oceanic regions exhibiting exceptionally high levels. This further exemplifies the importance of DMSP and its import as a nutrient and antistress compound for marine bacteria.

DMSP shares structural similarities with QACs. The DMSP binding mode of DmpX is similar with the reported binding modes of SBPs for QACs [42, 50–53], which can be summarized as an aromatic box formed by three or four aromatic residues to accommodate the quaternary amine, as well as two or three hydrophilic residues that form hydrogen bonds with the polar atoms of QACs [42, 50–53]. QACs contain a positive-charged nitrogen atom, similar to the positive-charged sulfur atom in DMSP. However, to the best of our knowledge, a hydrogen bond interaction between residues and the nitrogen atom of QACs have never been reported before. Thus, the hydrogen bond formed between Tyr200 and DMSP may represent a novel substrate binding and recognition pattern of SBPs.

To conclude, DMSP is an abundant organosulfur compound in the ocean, and plays important roles in marine stress responses, microbial food webs and the global sulfur and carbon cycling. Despite some bacteria producing DMSP themselves [4, 54–56], the uptake of DMSP is the prerequisite for most marine bacterial utilisation. Here, we identified an efficient ABC-type transporter DmpXWV, which is specific for DMSP and prevalent in numerous cosmopolitan marine bacteria. The DMSP-binding mode of DMSP in DmpX was also revealed. The results offer a better understanding of the global sulfur cycle driven by bacteria.

## Supplementary information

Supplementary figures

Table S3

Table S4

Table S5

Table S1

Table S2

## Data Availability

The structure of *Rn*DmpX/DMSP complex has been deposited in the PDB under the accession codes 7YLE.

## References

[1] Gali M, Devred E, Levasseur M, Royer SJ, Babin M (2015). A remote sensing algorithm for planktonic dimethylsulfoniopropionate (DMSP) and an analysis of global patterns. Remote Sens Environ.

[2] Zhang XH, Liu J, Liu JL, Yang GP, Xue CX, Curson ARJ (2019). Biogenic production of DMSP and its degradation to DMS-their roles in the global sulfur cycle. Sci China Life Sci.

[3] Alcolombri U, Ben-Dor S, Feldmesser E, Levin Y, Tawfik DS, Vardi A (2015). Identification of the algal dimethyl sulfide-releasing enzyme: A missing link in the marine sulfur cycle. Science.

[4] Curson AR, Liu J, Bermejo Martinez A, Green RT, Chan Y, Carrion O (2017). Dimethylsulfoniopropionate biosynthesis in marine bacteria and identification of the key gene in this process. Nat Microbiol.

[5] Sun J, Todd JD, Thrash JC, Qian Y, Qian MC, Temperton B (2016). The abundant marine bacterium *Pelagibacter* simultaneously catabolizes dimethylsulfoniopropionate to the gases dimethyl sulfide and methanethiol. Nat Microbiol.

[6] Zubkov MV, Fuchs BM, Archer SD, Kiene RP, Amann R, Burkill PH (2001). Linking the composition of bacterioplankton to rapid turnover of dissolved dimethylsulphoniopropionate in an algal bloom in the North Sea. Environ Microbiol.

[7] Curson AR, Todd JD, Sullivan MJ, Johnston AW (2011). Catabolism of dimethylsulphoniopropionate: microorganisms, enzymes, and genes. Nat Rev Microbiol.

[8] Vallina SM, Simó R (2007). Strong relationship between DMS and the solar radiation dose over the global surface ocean. Science.

[9] Suzuki MT, Beja O, Taylor LT, Delong EF (2001). Phylogenetic analysis of ribosomal RNA operons from uncultivated coastal marine bacterioplankton. Environ Microbiol.

[10] Luo H, Moran MA (2014). Evolutionary ecology of the marine *Roseobacter* clade. Microbiol Mol Biol Rev.

[11] Simon M, Scheuner C, Meier-Kolthoff JP, Brinkhoff T, Wagner-Dobler I, Ulbrich M (2017). Phylogenomics of *Rhodobacteraceae* reveals evolutionary adaptation to marine and non-marine habitats. ISME J.

[12] Buchan A, Gonzalez JM, Moran MA (2005). Overview of the marine *Roseobacter* lineage. Appl Environ Microbiol.

[13] Lidbury I, Murrell JC, Chen Y (2014). Trimethylamine *N*-oxide metabolism by abundant marine heterotrophic bacteria. Proc Natl Acad Sci USA.

[14] Tripp HJ, Kitner JB, Schwalbach MS, Dacey JW, Wilhelm LJ, Giovannoni SJ (2008). SAR11 marine bacteria require exogenous reduced sulphur for growth. Nature.

[15] Morris RM, Rappe MS, Connon SA, Vergin KL, Siebold WA, Carlson CA (2002). SAR11 clade dominates ocean surface bacterioplankton communities. Nature.

[16] Liu J, Xue CX, Wang J, Crombie AT, Carrion O, Johnston AWB (2022). *Oceanospirillales* containing the DMSP lyase DddD are key utilisers of carbon from DMSP in coastal seawater. Microbiome.

[17] Reisch CR, Moran MA, Whitman WB (2008). Dimethylsulfoniopropionate-dependent demethylase (DmdA) from *Pelagibacter ubique* and *Silicibacter pomeroyi*. J Bacteriol.

[18] Kiene RP, Williams LPH, Walker JE (1998). Seawater microorganisms have a high affinity glycine betaine uptake system which also recognizes dimethylsulfoniopropionate. Aquat Micro Ecol.

[19] Ziegler C, Bremer E, Kramer R (2010). The BCCT family of carriers: from physiology to crystal structure. Mol Microbiol.

[20] Todd JD, Curson ARJ, Nikolaidou-Katsaraidou N, Brearley CA, Watmough NJ, Chan YH (2010). Molecular dissection of bacterial acrylate catabolism - unexpected links with dimethylsulfoniopropionate catabolism and dimethyl sulfide production. Environ Microbiol.

[21] Sun L, Curson ARJ, Todd JD, Johnston AWB (2012). Diversity of DMSP transport in marine bacteria, revealed by genetic analyses. Biogeochemistry.

[22] Li CY, Wang XJ, Chen XL, Sheng Q, Zhang S, Wang P (2021). A novel ATP-dependent dimethylsulfoniopropionate lyase in bacteria that releases dimethyl sulfide and acryloyl-CoA. Elife.

[23] Davidson AL, Chen J (2004). ATP-binding cassette transporters in bacteria. Annu Rev Biochem.

[24] Rice AJ, Park A, Pinkett HW (2014). Diversity in ABC transporters: type I, II and III importers. Crit Rev Biochem Mol Biol.

[25] Cosquer A, Pichereau V, Pocard JA, Minet J, Cormier M, Bernard T (1999). Nanomolar levels of dimethylsulfoniopropionate, dimethylsulfonioacetate, and glycine betaine are sufficient to confer osmoprotection to *Escherichia coli*. Appl Environ Microbiol.

[26] Broy S, Chen CL, Hoffmann T, Brock NL, Nau-Wagner G, Jebbar M (2015). Abiotic stress protection by ecologically abundant dimethylsulfoniopropionate and its natural and synthetic derivatives: insights from *Bacillus subtilis*. Environ Microbiol.

[27] Teichmann L, Kummel H, Warmbold B, Bremer E (2018). OpuF, a new *Bacillus* compatible solute ABC transporter with a substrate-binding protein fused to the transmembrane domain. Appl Environ Microbiol.

[28] Noell SE, Giovannoni SJ (2019). SAR11 bacteria have a high affinity and multifunctional glycine betaine transporter. Environ Microbiol.

[29] Gonzalez JM, Covert JS, Whitman WB, Henriksen JR, Mayer F, Scharf B (2003). *Silicibacter pomeroyi* sp. nov. and *Roseovarius nubinhibens* sp. nov., dimethylsulfoniopropionate-demethylating bacteria from marine environments. Int J Syst Evol Microbiol.

[30] Moran MA, Buchan A, Gonzalez JM, Heidelberg JF, Whitman WB, Kiene RP (2004). Genome sequence of *Silicibacter pomeroyi* reveals adaptations to the marine environment. Nature.

[31] Reisch CR, Crabb WM, Gifford SM, Teng Q, Stoudemayer MJ, Moran MA (2013). Metabolism of dimethylsulphoniopropionate by *Ruegeria pomeroyi* DSS-3. Mol Microbiol.

[32] Todd JD, Kirkwood M, Newton-Payne S, Johnston AWB (2012). DddW, a third DMSP lyase in a model *Roseobacter* marine bacterium, *Ruegeria pomeroyi* DSS-3. ISME J.

[33] Smith AF, Rihtman B, Stirrup R, Silvano E, Mausz MA, Scanlan DJ (2019). Elucidation of glutamine lipid biosynthesis in marine bacteria reveals its importance under phosphorus deplete growth in *Rhodobacteraceae*. ISME J.

[34] Wirth JS, Wang T, Huang Q, White RH, Whitman WB (2020). Dimethylsulfoniopropionate sulfur and methyl carbon assimilation in *Ruegeria* species. mBio.

[35] Minor W, Cymborowski M, Otwinowski Z, Chruszcz M (2006). HKL-3000: the integration of data reduction and structure solution-from diffraction images to an initial model in minutes. Acta Crystallogr D Biol Crystallogr.

[36] Winn MD, Ballard CC, Cowtan KD, Dodson EJ, Emsley P, Evans PR (2011). Overview of the CCP4 suite and current developments. Acta Crystallogr D Biol Crystallogr.

[37] Emsley P, Lohkamp B, Scott WG, Cowtan K (2010). Features and development of Coot. Acta Crystallogr D Biol Crystallogr.

[38] Adams PD, Afonine PV, Bunkoczi G, Chen VB, Davis IW, Echols N (2010). PHENIX: a comprehensive Python-based system for macromolecular structure solution. Acta Crystallogr D Biol Crystallogr.

[39] Beale R, Airs R (2016). Quantification of glycine betaine, choline and trimethylamine *N*-oxide in seawater particulates: Minimisation of seawater associated ion suppression. Anal Chim Acta.

[40] Stirrup R, Mausz MA, Silvano E, Murphy A, Guillonneau R, Quareshy M, et al. Aminolipids elicit functional trade-offs between competitiveness and bacteriophage attachment in *Ruegeria pomeroyi*. ISME J. 2022; 10.1038/s41396-022-01346-0. 10.1038/s41396-022-01346-0 PMC9938194 36477724

[41] Marie D, Brussaard CPD, Thyrhaug R, Bratbak G, Vaulot D (1999). Enumeration of marine viruses in culture and natural samples by flow cytometry. Appl Environ Microbiol.

[42] Li CY, Chen XL, Shao X, Wei TD, Wang P, Xie BB (2015). Mechanistic insight into trimethylamine *N*-oxide recognition by the marine bacterium *Ruegeria pomeroyi* DSS-3. J Bacteriol.

[43] Murphy ARJ, Scanlan DJ, Chen Y, Adams NBP, Cadman WA, Bottrill A (2021). Transporter characterisation reveals aminoethylphosphonate mineralisation as a key step in the marine phosphorus redox cycle. Nat Commun.

[44] Villar E, Vannier T, Vernette C, Lescot M, Cuenca M, Alexandre A (2018). The Ocean Gene Atlas: exploring the biogeography of plankton genes online. Nucleic Acids Res.

[45] Milanese A, Mende DR, Paoli L, Salazar G, Ruscheweyh HJ, Cuenca M (2019). Microbial abundance, activity and population genomic profiling with mOTUs2. Nat Commun.

[46] Chen C, Malek AA, Wargo MJ, Hogan DA, Beattie GA (2010). The ATP-binding cassette transporter Cbc (choline/betaine/carnitine) recruits multiple substrate-binding proteins with strong specificity for distinct quaternary ammonium compounds. Mol Microbiol.

[47] Wang P, Chen XL, Li CY, Gao X, Zhu DY, Xie BB (2015). Structural and molecular basis for the novel catalytic mechanism and evolution of DddP, an abundant peptidase-like bacterial Dimethylsulfoniopropionate lyase: a new enzyme from an old fold. Mol Microbiol.

[48] Li CY, Zhang D, Chen XL, Wang P, Shi WL, Li PY (2017). Mechanistic insights into dimethylsulfoniopropionate lyase DddY, a new member of the cupin superfamily. J Mol Biol.

[49] Vila-Costa M, Simó R, Harada H, Gasol JM, Slezak D, Kiene RP (2006). Dimethylsulfoniopropionate uptake by marine phytoplankton. Science.

[50] Schiefner A, Breed J, Bosser L, Kneip S, Gade J, Holtmann G (2004). Cation-pi interactions as determinants for binding of the compatible solutes glycine betaine and proline betaine by the periplasmic ligand-binding protein ProX from *Escherichia coli*. J Biol Chem.

[51] Oswald C, Smits SHJ, Hoing M, Sohn-Bosser L, Dupont L, Le Rudulier D (2008). Crystal structures of the choline/acetylcholine substrate-binding protein ChoX from *Sinorhizobium meliloti* in the liganded and unliganded-closed states. J Biol Chem.

[52] Wolters JC, Berntsson RPA, Gul N, Karasawa A, Thunnissen AMWH, Slotboom DJ (2010). Ligand binding and crystal structures of the substrate-binding domain of the ABC transporter OpuA. PLoS One.

[53] Pittelkow M, Tschapek B, Smits SHJ, Schmitt L, Bremer E (2011). The Crystal structure of the substrate-binding protein OpuBC from *Bacillus subtilis* in complex with choline. J Mol Biol.

[54] Williams BT, Cowles K, Bermejo Martinez A, Curson ARJ, Zheng Y, Liu J (2019). Bacteria are important dimethylsulfoniopropionate producers in coastal sediments. Nat Microbiol.

[55] Peng M, Li CY, Chen XL, Williams BT, Li K, Gao YN (2022). Insights into methionine *S*-methylation in diverse organisms. Nat Commun.

[56] Li CY, Crack JC, Newton-Payne S, Murphy ARJ, Chen X-L, Pinchbeck BJ (2022). Mechanistic insights into the key marine dimethylsulfoniopropionate synthesis enzyme DsyB/DSYB. mLife.

