# Supplementary material for: Ubiquitous occurrence of a dimethylsulfoniopropionate ABC transporter in abundant marine bacteria
Source: ISME J. 2023 Jan 27;17(4):579–87. doi: 10.1038/s41396-023-01375-3 (PMC10030565; doi:10.1038/s41396-023-01375-3)
Supplement: Supplementary file 1 — Supplementary figures [file 41396_2023_1375_MOESM1_ESM.docx]

Fig. S1. *Ruegeria pomeroyi* DSS-3 wild-type, Δ*dmpXWV::Gm* mutant and complemented mutant (comp. Δ*dmpXWV::Gm*) formed a single population during flow cytometry analysis used for cell counts.





Fig. S2. ITC data for titrations of DMSP into recombinant *Rn*DmpX. ITC traces (top) and integrated binding isotherms (bottom) are shown.





Fig. S3. ITC data for titrations of DMSP into *Rn*DmpX Tyr200Ala and Tyr200Phe mutants. ITC traces (top) and integrated binding isotherms (bottom) are shown.


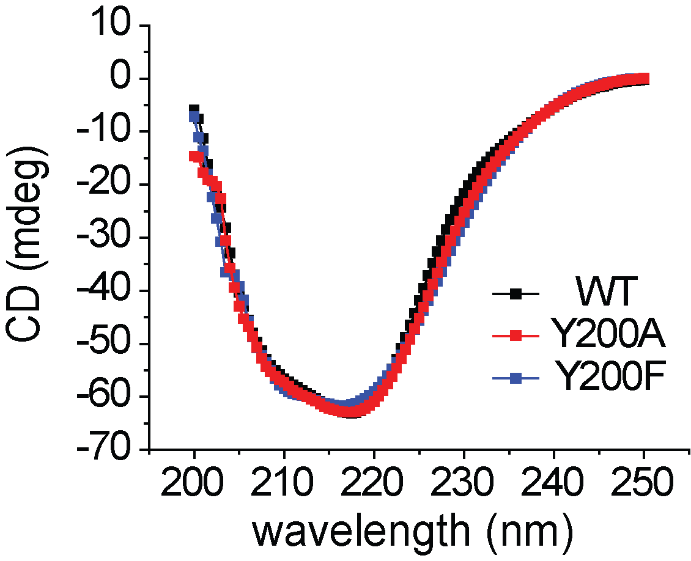


Fig. S4. CD spectra of WT *Rn*DmpX and its mutants.


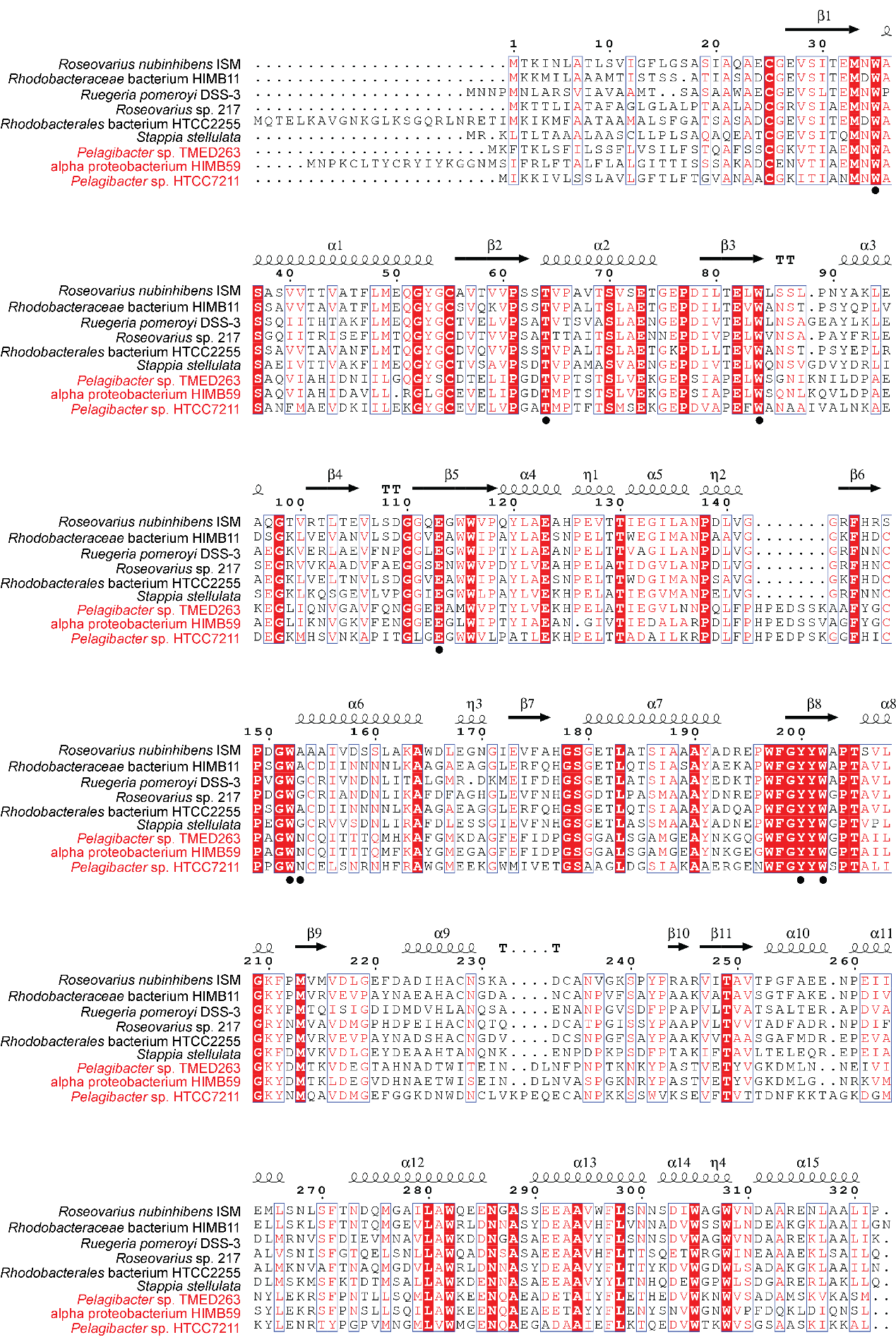


Fig. S5 Sequence alignment of DmpX proteins. Numbers in parentheses refer to the amino acid numbers in the DmpX sequence from *R.* *nubinhibens* ISM. DmpX proteins from strains of the MRG are coloured in black, and of the SAR11 clade in red. Residues involved in binding DMSP are marked with black dots.
