# Supplementary material for: Ubiquitous occurrence of a dimethylsulfoniopropionate ABC transporter in abundant marine bacteria
Source: ISME J. 2023 Jan 27;17(4):579–87. doi: 10.1038/s41396-023-01375-3 (PMC10030565; doi:10.1038/s41396-023-01375-3)
Supplement: Supplementary file 2 — Table S3 [file 41396_2023_1375_MOESM2_ESM.docx]

**Table S3.** Dissociation constant (*K_d_*) of DmpX of *Ruegeria pomeroyi* DSS-3 and SAR11 bacterium *Pelagibacter* sp. HTCC7211.

| *K_d_* | DMSP | GBT | choline | carnitine | TMAO |
| --- | --- | --- | --- | --- | --- |
| SPO2441 (DmpX) | 1.1 µM | - | - | - | - |
| DmpX^7211^ | 100 nM | - | - | - | - |

“-”, not detectable.
