# Supplementary material for: Ubiquitous occurrence of a dimethylsulfoniopropionate ABC transporter in abundant marine bacteria
Source: ISME J. 2023 Jan 27;17(4):579–87. doi: 10.1038/s41396-023-01375-3 (PMC10030565; doi:10.1038/s41396-023-01375-3)
Supplement: Supplementary file 3 — Table S4 [file 41396_2023_1375_MOESM3_ESM.docx]

**Table S4**. Uptake of ^14^C-DMSP by wild-type and mutant cultures.

| DMSP concentration [µM] | Uptake rate [fmol cell^-1^ min^-1^] | | |
| --- | --- | --- | --- |
|  | wild-type | 𝛥*dmpXWV*::Gm | complemented mutant 𝛥dmpXWV::Gm |
| 50 | 1.852 ± 0.100**** | 0.522 ± 0.040**** | 0.946 ± 0.299** |
| 150 | 2.267 ± 0.189**** | 0.823 ± 0.112**** | 1.221 ± 0.159* |
| 300 | 3.095 ± 0.081**** | 1.344 ± 0.150**** | 1.893 ± 0.084** |

* *p* < 0.05, ** *p* < 0.01, *** *p* < 0.0005, **** *p* < 0.0001

Values are means ± S.D. (n = 3).
