# Supplementary material for: Ubiquitous occurrence of a dimethylsulfoniopropionate ABC transporter in abundant marine bacteria
Source: ISME J. 2023 Jan 27;17(4):579–87. doi: 10.1038/s41396-023-01375-3 (PMC10030565; doi:10.1038/s41396-023-01375-3)
Supplement: Supplementary file 4 — Table S5 [file 41396_2023_1375_MOESM4_ESM.docx]

**Table S5.** Crystallographic data collection and refinement parameters of *Rn*DmpX.

| Parameters | *Rn*DmpX/DMSP complex  soaked with I3C | *Rn*DmpX/DMSP complex |
| --- | --- | --- |
| **Diffraction data** |  |  |
| Space group | *P*4_1_ | *P*4_1_ |
| Unit cell |  |  |
| a, b, c (Å) | 75.2, 75.2, 88.3 | 74.3, 74.3, 87.8 |
| α, β, γ (°) | 90.0, 90.0, 90.0 | 90.0, 90.0, 90. 0 |
| Resolution range (Å) | 50.0-2.5 (2.59-2.50) * | 50.0-2.05 (2.12-2.05) |
| Redundancy | 17.1 (17.9) | 7.5 (7.5) |
| Completeness (%) | 100.0 (100.0) | 100.0 (100.0) |
| *R*_merge_** | 0.1 (0.5) | 0.1 (0.5) |
| *I*/σ*I* | 26.6 (6.2) | 39.1 (4.6) |
| **Refinement statistics** |  |  |
| R-factor |  | 0.19 |
| Free R-factor |  | 0.22 |
| RMSD from ideal geometry |  |  |
| Bond lengths (Å) |  | 0.007 |
| Bond angles (°) |  | 1.05 |
| Ramachandran plot (%) |  |  |
| Favoured |  | 97.0 |
| Allowed |  | 3.0 |
| Overall B-factors (Å^2^) |  | 38.6 |

*Numbers in parentheses refer to data in the highest resolution shell.

***R*_merge_=∑*_hkl_*∑*_i_*|*I*(*hkl*)*_i_* -<*I*(*hkl*)>|/∑*_hkl_*∑*_i_I*(*hkl*)*_i_*, where *I* is the observed intensity, <*I*(*hkl*)> represents the average intensity, and *I*(*hkl*)*_i_* represents the observed intensity of each unique reflection.
